# Cement-Based Thermoelectric Device for Protection of Carbon Steel in Alkaline Chloride Solution

**DOI:** 10.3390/ma15134461

**Published:** 2022-06-24

**Authors:** Tao Ji, Xiao Liao, Shiping Zhang, Yan He, Xiaoying Zhang, Xiong Zhang, Weihua Li

**Affiliations:** 1 School of Architecture and Civil Engineering, Nanjing Institute of Technology, Nanjing 211167, China; 2 College of Civil Engineering, Qingdao University of Technology, Qingdao 266033, China; 18306519700@163.com (X.L.); zhangxy@qibebt.ac.cn (X.Z.); 3 School of Civil Engineering, Suzhou University of Science and Technology, Suzhou 215011, China; hey1019@sina.com; 4 School of Materials Science and Engineering, Tongji University, Shanghai 201804, China; 56zhangxiong@tongji.edu.cn; 5 School of Chemical Engineering and Technology, Sun Yat-sen University, Zhuhai 519082, China; yongjuan60@sina.com

**Keywords:** PANI/MnO_2_ composite, carbon steel, thermoelectric device, electrochemical techniques, cement-based materials

## Abstract

The thermoelectric cement-based materials can convert heat into electricity; this makes them promising candidates for impressed current cathodic protection of carbon steel. However, attempts to use the thermoelectric cement-based materials for energy conversion usually results in low conversion efficiency, because of the low electrical conductivity and Seebeck coefficient. Herein, we deposited polyaniline on the surface of MnO_2_ and fabricated a cement-based thermoelectric device with added PANI/MnO_2_ composite for the protection of carbon steel in alkaline chloride solution. The nanorod structure (70~80 nm in diameter) and evenly dispersed conductive PANI provide the PANI/MnO_2_ composite with good electrical conductivity (1.9 ± 0.03 S/cm) and Seebeck coefficient (−7.71 × 10^3^ ± 50 μV/K) and, thereby, increase the Seebeck coefficient of cement-based materials to −2.02 × 10^3^ ± 40 μV/K and the electrical conductivity of cement-based materials to 0.015 ± 0.0003 S/cm. Based on this, the corrosion of the carbon steel was delayed after cathodic protection, which was demonstrated by the electrochemical experiment results, such as the increased resistance of the carbon steel surface from 5.16 × 10^2^ Ω·cm^2^ to 5.14 × 10^4^ Ω·cm^2^, increased charge transfer resistance from 11.4 kΩ·cm^2^ to 1.98 × 10^6^ kΩ·cm^2^, and the decreased corrosion current density from 1.67 μA/cm^2^ to 0.32 μA/cm^2^, underlining the role of anti-corrosion of the PANI/MnO_2_ composite in the cathodic protection system.

## 1. Introduction

Reinforced concrete is the most widely used material in marine engineering, but chloride ions migrate into porous concrete from the marine environment and cause steel corrosion. This problem would lead to the deterioration and failure of reinforced concrete structures and ultimately reduce the safe service of marine engineering [1,2]. Cathodic protection proves to be an effective method for protecting the reinforced concrete vulnerable to chloride pollution [3]. However, a series of problems, such as the consumption of metal materials, the waste of electric energy, and environmental pollution, would take place in traditional cathodic protection [4,5].

To solve the above problems, solar cells, closed-cycle steam generators, thermoelectric generators, and other sustainable energy sources have been applied in cathodic protection [6,7,8]. Thermoelectric generators made of BiSb, Bi_2_Te_3_, PbTe, SiGe, and other alloys can convert heat into electricity when there is a temperature difference. However, these traditional thermoelectric generators are detached from the reinforced concrete structure. Adding the thermoelectric components, such as BiSb, Bi_2_Te_3_, PbTe, and SiGe, to cement paste would provide the cement-based materials with thermoelectric properties. Based on this, cement-based thermoelectric devices can be fabricated for protecting the carbon steel in alkaline chloride solution. However, the use of cement-based thermoelectric devices for cathodic protection usually results in low anti-corrosion efficiency, because of the low thermoelectric efficiency [9]. It is mainly increased by increasing the Seebeck coefficient and electrical conductivity for cement-based materials, as the thermal conductivity of cement-based materials is relatively stable.

To increase the Seebeck coefficient of the cement-based materials, adding carbon materials and metallic compounds are the main methods. Among the carbon materials, carbon fibers and modified carbon fibers were the early reported thermoelectric components [9,10,11,12]. For instance, the concrete increased the Seebeck coefficient to 250 μV/K by designing a pyrolytic carbon layer on the carbon fiber surface [12]. Other new carbon materials, including carbon nanotubes, expanded graphite, and graphene, were incorporated into the cement-based materials to increase the Seebeck coefficient [13,14,15,16,17,18]. For example, Tzounis et al. obtained a Seebeck coefficient of −880 μV/K by adding n-type nitrogen-doped CNTs [14]. Ghosh et al. obtained a Seebeck coefficient of 141.5 μV/K by adding 10 wt.% graphene and ZnO [16]. Wei et al. obtained a Seebeck coefficient of −54.5 μV/K with 5 wt.% expanded graphite at approximately 65 °C [18]. However, the absolute value of the Seebeck coefficient of cement-based materials with these conductive thermoelectric components has difficulty going beyond 1000 μV/K. Metallic compounds, including Bi_2_Te_3_, Ca_3_Co_4_O_9_, Fe_2_O_3_, Bi_2_O_3_, ZnO, and MnO_2_, were also used to increase the Seebeck coefficient of the cement-based materials [19,20,21,22,23]. For example, Wei et al. obtained a Seebeck coefficient of 100.28 μV/K by adding 5 wt.% Bi_2_O_3_ powder to the cement paste [20]. Recently, a high Seebeck coefficient of −3085 μV/K was obtained by incorporating 5 wt.% nanostructured MnO_2_ powder into the cement paste in our previous study [23]. The significantly increased Seebeck coefficient was mainly caused by the increased density of states of nanoparticles and increased interfaces in the hardened cement matrix [24,25,26,27]. However, these cement-based materials with large Seebeck coefficients exhibit low electrical conductivity, because of the low electrical conductivity of the semiconductor powders [23].

To increase the electrical conductivity of the cement-based materials, adding conductive particles and fibers, including inorganic and organic materials, are the main methods [28,29,30,31,32]. Carbon fiber, acting as one of the most widely used conductive fibers, increased the electrical conductivity of the cement composites to 0.005 S/cm when the volume fraction of carbon fiber exceeded 0.8% [28]. When the volume fraction of cut virgin carbon fibers, recycled carbon fibers, and brass-plated steel fibers exceeded 0.8% in the cement matrix, fibers would form an effective conductive network, thus increasing the electrical contact bridges [29]. Conductive polymers are also commonly used in cement-based materials to increase their electrical conductivity [33,34,35,36]. Among them, conductive polyaniline is the most widely used due to its easy availability, stable chemical properties, and high conductivity [37]. When the volume content of polyaniline exceeded 1.0%, a good network acting as a conductive channel could be formed in the cement matrix, according to the classical percolation theory [30]. However, these conductive fillers cannot result in a significant increment in the Seebeck coefficient of the cement-based materials. Considering the significantly increased Seebeck coefficient by nanostructured MnO_2_ and increased electrical conductivity by polyaniline, it is interesting to see if the polyaniline/manganese dioxide composite can significantly increase the Seebeck coefficient and electrical conductivity of cement-based materials.

Herein, we designed and fabricated a cement-based thermoelectric device with a synthesized polyaniline/manganese dioxide composite inside. It is expected that the cement-based thermoelectric device can exhibit a good Seebeck coefficient and electrical conductivity and, thus, offer a sufficient cathodic current for the carbon steel in chloride-polluted simulated concrete pore solution under a temperature difference. Based on the experiments, the increased thermoelectric effect of cement-based materials and the cathodic protection effect were discussed in detail. To our knowledge, cement-based thermoelectric devices and their application in cathodic protection for carbon steels in simulated concrete pore solution were studied for the first time.

## 2. Materials and Methods

### 2.1. Synthesis of PANI/MnO_2_ Composite and Cement-Matrix Composites Containing the PANI/MnO_2_ Composite

Aniline (ANI), ammonium persulfate, hydrochloric acid, and manganese dioxide were used to prepare the PANI/MnO_2_ composite. Aniline and nanostructured manganese dioxide were purchased from Macklin Biochemical Co., Ltd., Shanghai. Ammonium persulfate and hydrochloric acid were purchased from Sinopharm Chemical Reagent Co., Ltd., Shanghai. Hydrochloric acid was diluted to obtain a concentration of 1 mol/L before use. The No. 1 solution (S1) was obtained by mixing 0.9313 g aniline in 25 mL HCl solution, and the No. 2 solution (S2) was obtained by mixing 2.2820 g ammonium persulfate in 25 mL HCl solution at 25 °C. Then, S1 was added to S2 drop by drop. After that, manganese dioxide powder (4.0, 6.0, 8.0, 10.0, and 15.0 g) was slowly added into the mixture and magnetically stirred for 6 h. The received products were washed 10 times and dried at 60 °C for 24 h, and they were named C1, C2, C3, C4, and C5 for 4.0, 6.0, 8.0, 10.0, and 15.0 g manganese dioxide powder in the reaction, respectively.

The synthesized PANI/MnO_2_ composite was characterized by Fourier transform infrared (FTIR) spectroscopy, scanning electron microscopy (SEM), and thermogravimetric analysis (TGA). FTIR pattern measurements were carried out by an FT-IR spectrometer (Tensor 27, Bruker, Karlsruhe, Germany) with a wavelength range of 4000 to 400 cm^−1^ and a resolution of 4 cm^−1^. SEM images were acquired by secondary electrons (SE) with a tabletop microscope (S-3400, Hitachi, Tokyo, Japan) with a 15 kV acceleration voltage. TGA was conducted with a thermogravimetric analyzer (1100SF, Mettler Toledo, Zurich, Switzerland) by heating the sample from ambient temperature to 800 K at a rate of 10 K/min.

Calcium sulphoaluminate cement, fly ash, water, superplasticizer, and the synthesized PANI/MnO_2_ powder (C2) were used to make the cement-matrix composites. The mixing proportion of the cement paste is listed in Table 1.

### 2.2. Thermoelectric Properties of PANI/MnO_2_ Composite and Cement-Matrix Composites Containing the PANI/MnO_2_ Composite

**Figure 1:**
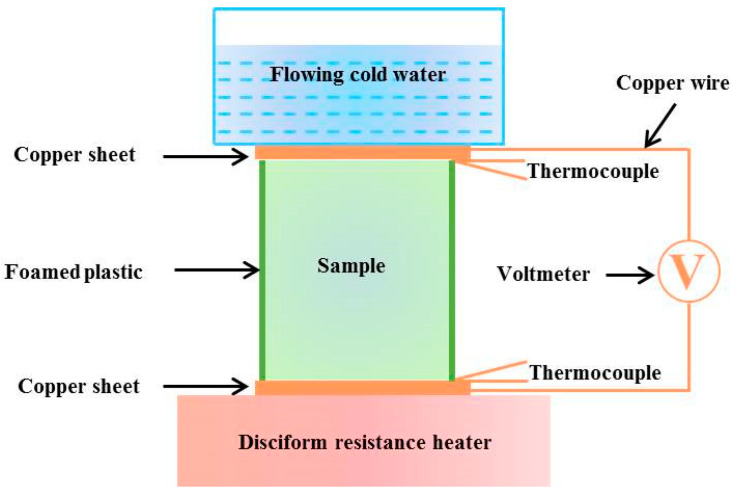
The schematic of the apparatus for measuring Seebeck coefficient around room temperature.

The electrical conductivity of the PANI/MnO_2_ composite and cement-matrix composites containing the PANI/MnO_2_ composite was measured by a four-probe conductivity tester (FT-301, Rooko, Ningbo, China) at ambient temperature, where two copper plates adhered to the sample sides worked as current contacts and two copper meshes inserted into the sample worked as voltage connects. Thus, the electrical conductivity can be calculated by the obtained current, voltage, and sizes of the PANI/MnO_2_ composite or the cement-matrix composites containing the PANI/MnO_2_ composite sample. The apparatus and method used to measure the Seebeck coefficient of the PANI/MnO_2_ composite or the cement-matrix composites containing the PANI/MnO_2_ composite sample were the same as those used for MnO_2_ powder in our previous study, as shown in Figure 1 [23]. The PANI/MnO_2_ composite was added into a special plastic pipe and pressed to 5.0 MPa. The cement-matrix composites were grinded with emery papers (grade 100 and 600) to make the surfaces flat. The copper sheets and the sample were connected naturally by the gravity of the water tank on the top of the sample. The applied pressure of the water tank was approximately 2.5 MPa. In the apparatus, one copper sheet at the end of the sample was connected to a disciform resistance heater (0.05 K/s) and another copper sheet at the end was connected to flowing cold water. In addition, the two copper sheets were connected to a Fluke 289 C multimeter and two Type K thermocouples by copper wires to test the Seebeck voltage and temperature difference, respectively. Each determination was conducted five times to avoid accidental error.

### 2.3. Preparation of Cement-Based Thermoelectric Device for Cathodic Protection

**Figure 2:**
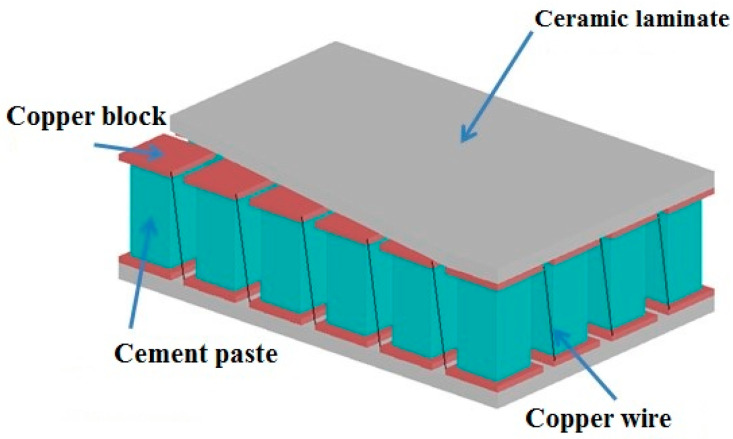
Schematic diagram of the cement-based thermoelectric device.

The cement-based thermoelectric device mainly included cement-paste blocks (M6), copper blocks, copper wires, and ceramic laminates, as shown in Figure 2. In the device, 24 hardened cement-paste blocks (40 × 40 × 160 mm) were connected in series by copper wire. A layer of silicone grease (3875, SINWE, Shenzhen, China) was applied between the cement-paste block and the copper block to reduce the thermal resistance. In addition, the side of the cement-paste block was covered with a layer of foam insulation to reduce heat loss during heat transfer.

**Figure 3:**
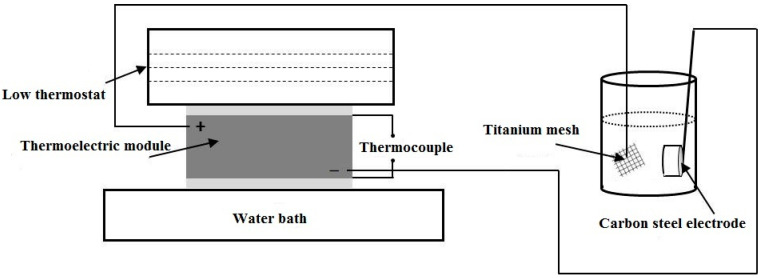
Schematic diagram of the cathodic protection system.

The cathodic protection system mainly included a cement-based thermoelectric device, low thermostat, water bath, thermocouple, titanium mesh, and carbon steel electrode, as shown in Figure 3. A water bath, low thermostat, and thermocouple were used to maintain a temperature difference of 20 K between the two opposite surfaces of the cement-based thermoelectric device. The positive pole and negative pole were connected to the titanium mesh and carbon steel electrode, respectively. The carbon steel electrode was prepared by welding a carbon steel block (1.00 × 1.00 × 1.00 cm) to a copper wire. Five surfaces of the carbon steel block were coated by epoxy resin, and the rest of the surface was ground with emery paper (grade 1000). The titanium mesh and carbon steel electrode were immersed in the solution. Thus, the carbon steel electrode can be protected by the impressed current from the thermoelectric module. The simulated concrete pore solution was obtained by diluting an alkaline solution (0.6 mol/L KOH + 0.2 mol/L NaOH + 0.01 mol/L Ca(OH)_2_) until the pH decreased to 12.5. To simulate the corrosion condition of seawater, 3.5 wt.% NaCl was added into the alkaline solution. The 3.5 wt.% NaCl-polluted simulated concrete pore solution was named NSCS.

### 2.4. Electrochemical Experiments

The open circuit potential (OCP), electrochemical impedance spectroscopy (EIS), and polarization potentiodynamics (PP) of the carbon steel electrode were tested with a PARSTAT 2273 potentiostat/galvanostat in NSCS. A saturated calomel electrode and a platinum slice were immersed in the solution and used as the reference electrode and counter electrode, respectively.

The OCP of the carbon steel electrode was carried out once the cathodic protection was suspended and stopped when the potential did not change by more than 2 mV in 300 s. The EIS of the carbon steel electrode was carried out with sine wave circuit excitation (amplitude: 10 mV; frequency: 10^5^–10^−2^ Hz). After that, the PP measurement was carried out with a scan rate of 1 mV s^−1^ from −250 mV to +250 mV versus the obtained potential from OCP.

## 3. Results and Discussion

### 3.1. Characterizations of PANI/MnO_2_ Composite

**Figure 4:**
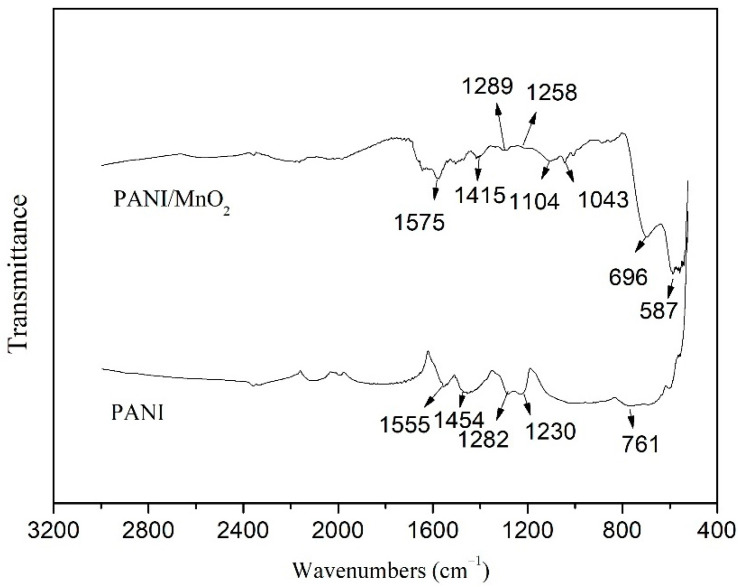
Fourier transform infrared transmission spectra of PANI and PANI/MnO_2_ composite (C2).

The PANI/MnO_2_ powders prepared with five different dosages of MnO_2_ were characterized by FT-IR. The obtained results showed that the dose of MnO_2_ powder had no effect on the location of the characteristic absorption peaks. Therefore, any one of the five synthesized products can be used to analyze the structure. The FT-IR transmission spectra of the PANI and PANI/MnO_2_ composite (C2) are shown in Figure 4. In the spectra of PANI, the absorption peaks near 1555 cm^−1^ and 1454 cm^−1^ correspond to the stretching vibrations of C=C in the quinone ring and benzene ring, respectively. The absorption peak near 761 cm^−1^ corresponds to the bending vibration of the C-H out-of-plane. The absorption peaks near 1282 cm^−1^ and 1230 cm^−1^ correspond to the stretching vibrations of C-N [38]. Compared with the spectra of PANI, the positions of the absorption peaks corresponding to C=C, C-N, and C-H in the spectra of the PANI/MnO_2_ composite shift slightly, which is caused by MnO_2_ [39]. The absorption peaks near 1104 and 1043 cm^−1^ correspond to O-H stretching vibration caused by the H_2_O in the voids formed by Mn and O atoms. In addition, an absorption peak near 587 cm^−1,^ corresponding to the bending vibration of Mn-O, is found in the spectra of the PANI/MnO_2_ composite [39]. The reaction product is identified as a PANI/MnO_2_ composite from the FT-IR results.

**Figure 5:**
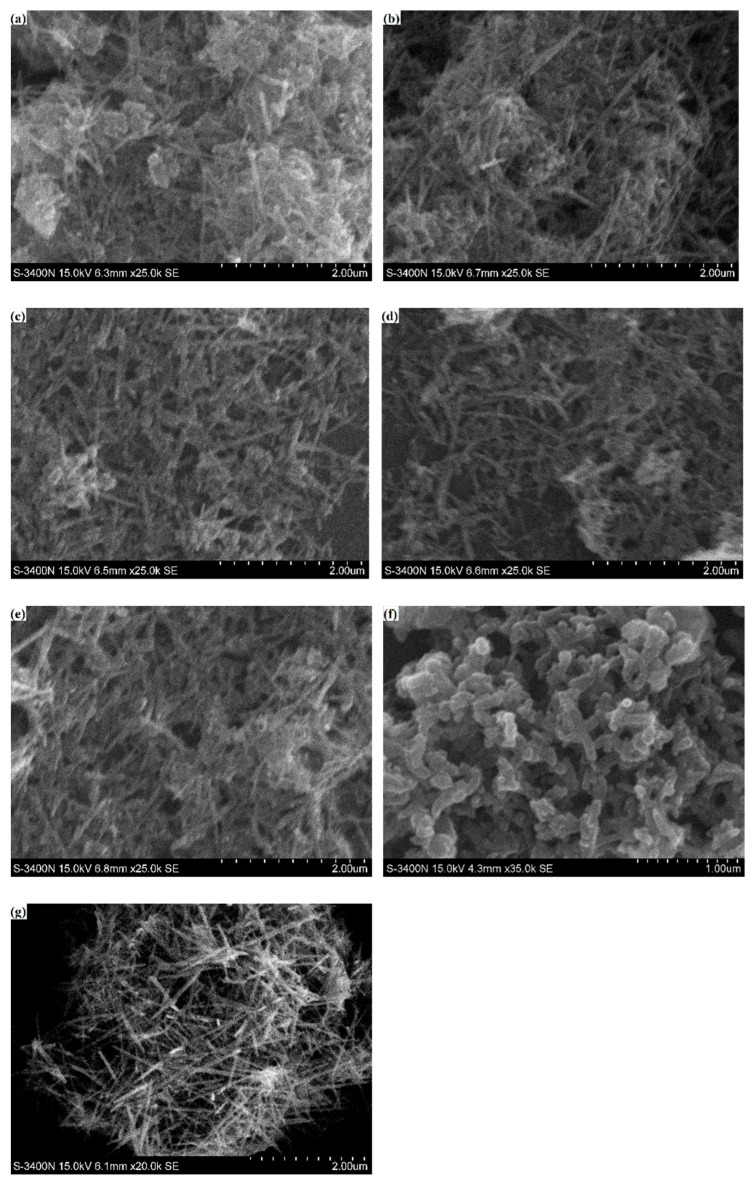
SEM-SE morphologies of PANI/MnO_2_ composites ((**a**): C1; (**b**): C2; (**c**): C3; (**d**): C4; (**e**): C5), pure PANI (**f**) and pure MnO_2_ (**g**).

Figure 5 shows the SEM images of the synthesized PANI/MnO_2_ composite, PANI, and MnO_2_. Among them, a, b, c, d, and e are the SEM images of C1, C2, C3, C4, and C5, respectively; f is the SEM image of pure PANI powder prepared without MnO_2_; g is the SEM image of pure MnO_2_ powder. It can be found that the microstructure of the five synthesized PANI/MnO_2_ composites were a mixture of rods and clusters and some clusters attached around the rods and cross-linked with each other. The purchased MnO_2_ powders are rod shaped with a diameter of approximately 70 nm and a length of approximately 1.2 μm. The rod shape is the typical morphology of MnO_2,_ and the cluster shape is the typical morphology of PANI according to previous study [23,38]. The reaction product was identified as a PANI/MnO_2_ composite from the SEM results. In addition, some separated clusters appeared in C1, and some separated rods appeared in C5. This phenomenon indicates that a uniform PANI/MnO_2_ morphology cannot be obtained when the dose of MnO_2_ is out of a reasonable range in the reaction. In the polymerization reaction of aniline, the MnO_2_ nanorod worked as the substrate. Therefore, the PANI coated the MnO_2_ nanorods first and distributed among the PANI/MnO_2_ nanorods when it was abundant, according to the SEM images. The rods in the images exhibit a diameter of approximately 70~80 nm, indicating that the synthesized PANI/MnO_2_ composite is nanostructured.

**Figure 6:**
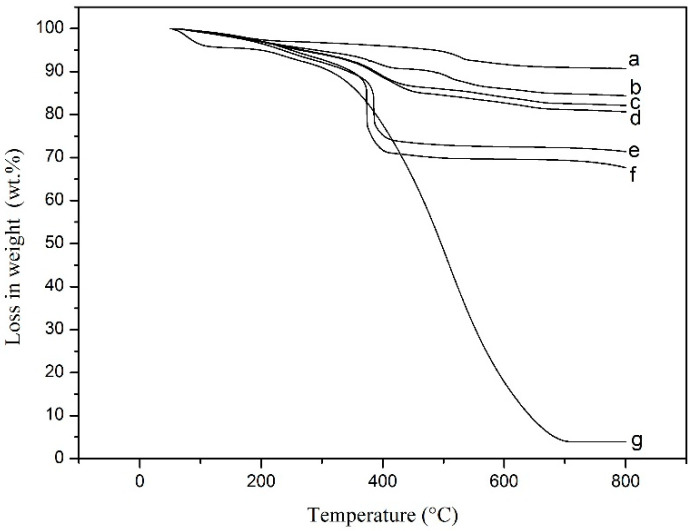
TGA curves of pure MnO_2_ (a), PANI/MnO_2_ composites (b: C1; c: C2; d: C3; e: C4; f: C5), and pure PANI (g).

Figure 6 shows the TGA curves of pure MnO_2_, the PANI/MnO_2_ composites, and pure PANI. Pure MnO_2_ exhibits a weight loss of 2.6% from 50 to 200 °C due to water evaporation and a weight loss of 6.7% from 200 to 800 °C due to oxygen release, as MnO_2_ changes to Mn_2_O_3_ under heating [39,40]. Pure PANI exhibits a weight loss of 5.0% from 50 to 200 °C due to water evaporation and a weight loss of 91% from 200 to 718 °C due to organic decomposition [39,41]. The residues of pure MnO_2_ and pure PANI were 90.7% and 4.0%, respectively. C1, C2, C3, C4, and C5 exhibit a weight loss of water from 50 to 200 °C, and a weight loss from 200 to 800 °C due to oxygen release and organic decomposition, and leave 67.7%, 71.4%, 80.6%, 82.1%, and 84.4% residue, respectively. Based on the residue of pure MnO_2_, the PANI/MnO_2_ composites, and pure PANI, the contents of PANI and MnO_2_ in C1, C2, C3, C4, and C5 can be calculated, as shown in Table 2. It shows that the content of MnO_2_ in the prepared PANI/MnO_2_ composite increases with increasing doses of MnO_2_ in the reaction. Some MnO_2_ added in the reaction system would participate in the redox reaction as follows [39,42]:

(1) $$2H^{+}+MnO_{2}+ANI\to PANI+2H_{2}O+Mn^{2+}$$

where MnO_2_ acts as an oxidant and ANI acts as a reducing agent. Some MnO_2_ turns into soluble Mn^2+^, which reduces the theoretical MnO_2_ content in the PANI/MnO_2_ composite.

### 3.2. Thermoelectric Properties of PANI/MnO_2_ Composite and Cement-Matrix Composites Containing the PANI/MnO_2_ Composite

**Figure 7:**
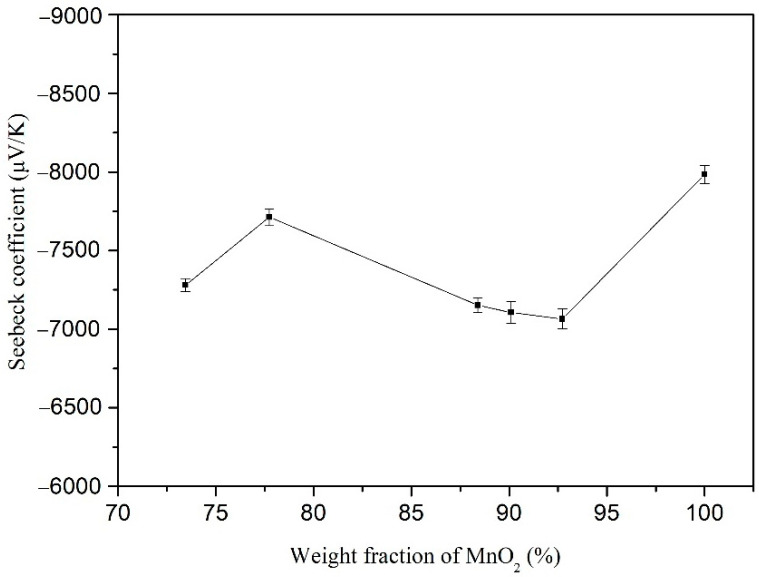
Seebeck coefficient of PANI/MnO_2_ composite with different weight fractions of MnO_2_ and pure MnO_2._

Figure 7 shows the Seebeck coefficient of the PANI/MnO_2_ composite with different weight fractions of MnO_2_ and pure MnO_2_. The Seebeck coefficient of the prepared PANI/MnO_2_ composites is lower than that of pure MnO_2_, indicating that MnO_2_ contributes more to the Seebeck coefficient than PANI in the composite. The Seebeck coefficient of the PANI/MnO_2_ composite reaches the maximum value of −7.72 × 10^3^ ± 50 μV/K when the weight fraction of MnO_2_ in the composite is 77.7%. The obtained maximum Seebeck coefficient was less than that of compacted flake-shaped β-MnO_2_ powders (20,000–40,000 μV/K) from Song et al. [42]. It may be caused by the difference in structure, size distribution, and morphology of the MnO_2_ particles. The Seebeck coefficient of the PANI/MnO_2_ composite reaches the minimum value of −7.06 × 10^3^ ± 60 μV/K when the weight fraction of MnO_2_ in the composite is 92.7%. When the weight fraction of MnO_2_ in the composite exceeds 77.7%, the Seebeck coefficient of the PANI/MnO_2_ composite gradually decreases with the increasing weight fraction of MnO_2_. This changing trend may be caused by the increased pores in the compacted PANI/MnO_2_ composite powder, as the particle uniformity decreased with increasing the MnO_2_ fraction [42].

**Figure 8:**
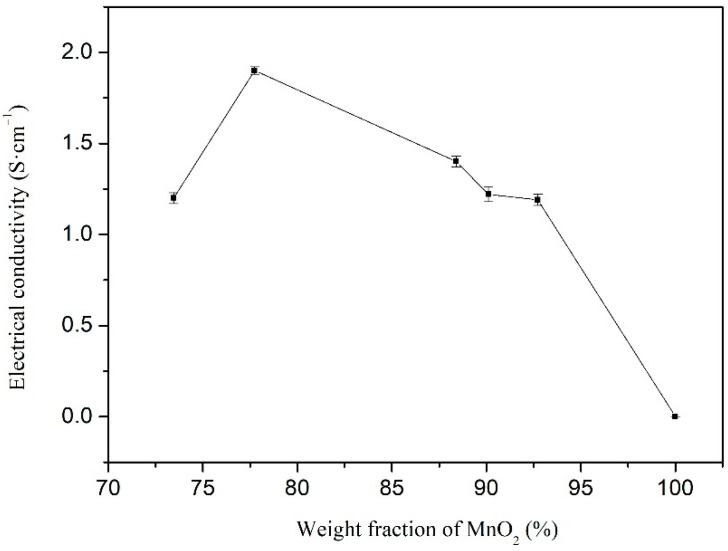
Electrical conductivity of PANI/MnO_2_ composite with different weight fractions of MnO_2_ and pure MnO_2._

Figure 8 shows the electrical conductivity of the PANI/MnO_2_ composite with different weight fractions of MnO_2_ and pure MnO_2_. Compared with the PANI/MnO_2_ composite, the electrical conductivity of pure MnO_2_ is negligible, indicating that PANI is the main conductivity source of PANI/MnO_2_. The electrical conductivity of the PANI/MnO_2_ composite fluctuates between 1.2 ± 0.03 and 1.9 ± 0.03 S/cm. The electrical conductivity of the PANI/MnO_2_ composite decreases with increasing the weight fraction of MnO_2_ when it is high. In the PANI/MnO_2_ composite, PANI serves as a fast conductive path for electron transport, so it is easy to see that the electrical conductivity decreases with increasing the MnO_2_ content [43]. The electrical conductivity of the PANI/MnO_2_ composite increases with increasing the weight fraction of MnO_2_ when it is low. It may be caused by the aggregation of PANI particles according to the SEM image (Figure 5a). The PANI cluster in the PANI/MnO_2_ composite increases the conduction path in the compacted powder for testing. In addition, the PANI cluster increased pores in the compacted PANI/MnO_2_ composite powder, resulting in the decrease of conduction channels [42]. When the weight fraction of MnO_2_ in the composite was 77.7%, the electrical conductivity of the PANI/MnO_2_ composite reached a maximum value of 1.9 ± 0.03 S/cm. The obtained Seebeck coefficient and electrical conductivity of the materials in this work are similar to the composites from Wei, Sampad Ghosh, and some other researchers [44,45,46]. The Seebeck coefficient and electrical conductivity of those compacted composites with complex conductive “networks” inside are higher than those of the simple composite system, such as the unidirectional fibre-reinforced composites [47].

**Figure 9:**
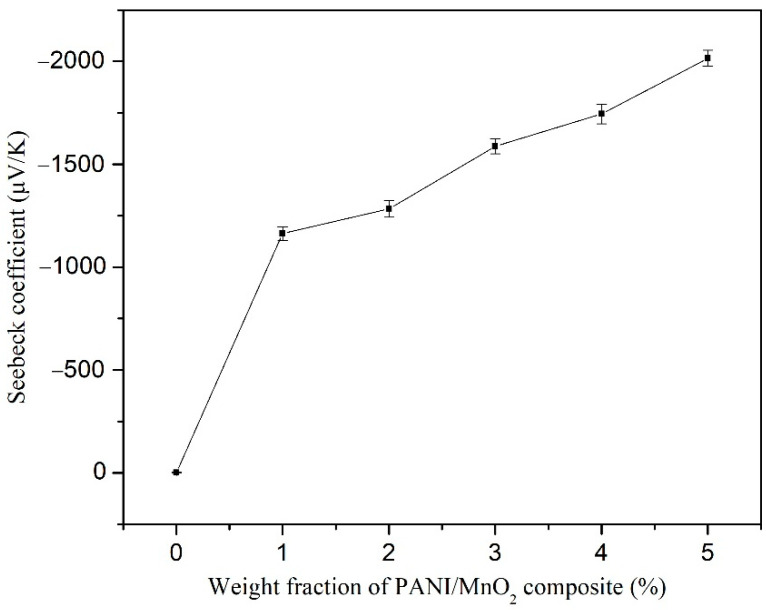
Seebeck coefficient of the cement-matrix composites containing different dosages of PANI/MnO_2_ composite.

**Figure 10:**
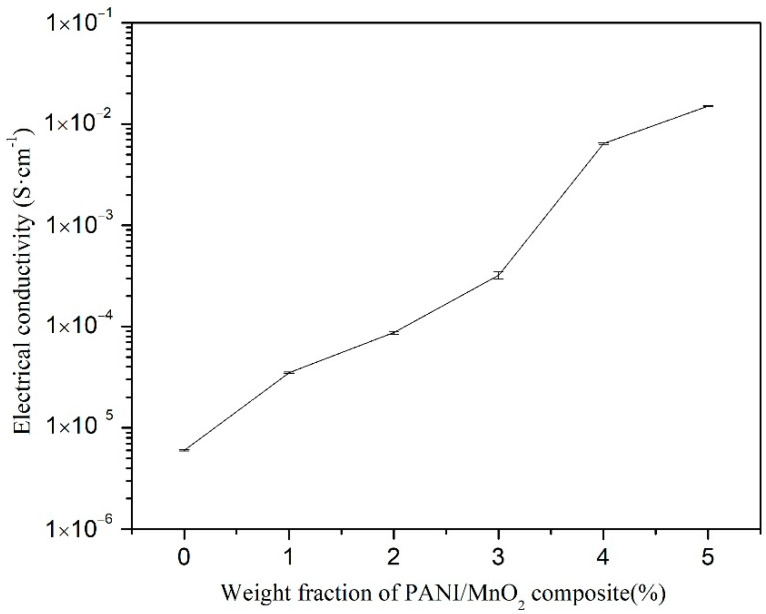
Electrical conductivity of the cement-matrix composites containing different dosages of PANI/MnO_2_ composite.

Due to the Seebeck coefficient and electrical conductivity results, the PANI/MnO_2_ composite (C2) was used as the thermoelectric component in the cement-matrix composites. The Seebeck coefficient and electrical conductivity of the cement-matrix composites containing different dosages of PANI/MnO_2_ composite are shown in Figure 9 and Figure 10, respectively. The Seebeck coefficient and electrical conductivity of the cement-matrix composites increases with increasing the dose of PANI/MnO_2_ composite. When the PANI/MnO_2_ composite content was 5.0 wt.% of the cement, the electrical conductivity of the cement-matrix composites reached a maximum value of 0.015 ± 0.0003 S/cm, and the maximum Seebeck coefficient was −2.02 × 10^3^ ± 40 μV/K. The obtained maximum Seebeck coefficient was higher than the reported values, such as −880 μV/K of cement composites with 1 wt.% expanded n-type nitrogen-doped CNTs, 746 μV/K of cement composites with 3 wt.% expanded graphite, 168.12 μV/K of cement composites with 1 wt.% reduced graphene oxide, and 36.3 μV/K of cement composites with 0.45 wt.% Bi_2_Te_3_ [14,44,48,49]. It is mainly caused by the rod-shaped PANI/MnO_2_ composite, acting as a one-dimensional nanostructured material. The decreased dimension would dramatically increase the gradient of the density of states relative to the energy near the Fermi energy and increase the Seebeck coefficient finally [48]. However, it was less than that of cement composites with 5.0 wt.% MnO_2_ powder (−3085 μV/K), which was caused by the low Seebeck coefficient of PANI [23]. The corresponding electrical conductivity maximum of the cement-matrix composites with 5.0 wt.% PANI/MnO_2_ composite reached the maximum value (0.015 ± 0.0004 S/cm). At this content, PANI covered 1.91% of the volume of the cement matrix, resulting in a good conductive network according to the classical percolation theory [40]. The obtained maximum electrical conductivity was 10,000 times that of plain cement paste, 80 times that of cement composites with 5.0 wt.% MnO_2_ powder, and it was higher than the reported values, such as 0.0064 S/cm cement composites with 3 wt.% expanded graphite, 0.0027 S/cm of cement composites with 1 wt.% reduced graphene oxide, 0.0011 S/cm of cement composites with 0.45 wt.% Bi_2_Te_3_, and a bit lower than the cement composites with 1 wt.% expanded n-type nitrogen-doped CNTs (0.0195 S/cm) [14,44,48,49]. The thermoelectric effect of the cement-matrix composites with added PANI/MnO_2_ composite was more remarkable than that of those cement composites with added CNTs, expanded graphite, reduced graphene oxide, or Bi_2_Te_3_. In the cement-matrix composites with added PANI/MnO_2_ composite, PANI contributes more to electrical conductivity and contributes less to the Seebeck coefficient than MnO_2_. Then, the cement-matrix composites with 5.0 wt.% or 2.98 vol.% PANI/MnO_2_ composite were used to fabricate a cement-based thermoelectric device for cathodic protection with a current density of 0.41 mA/cm^2^.

### 3.3. Open Circuit Potential (OCP)

**Figure 11:**
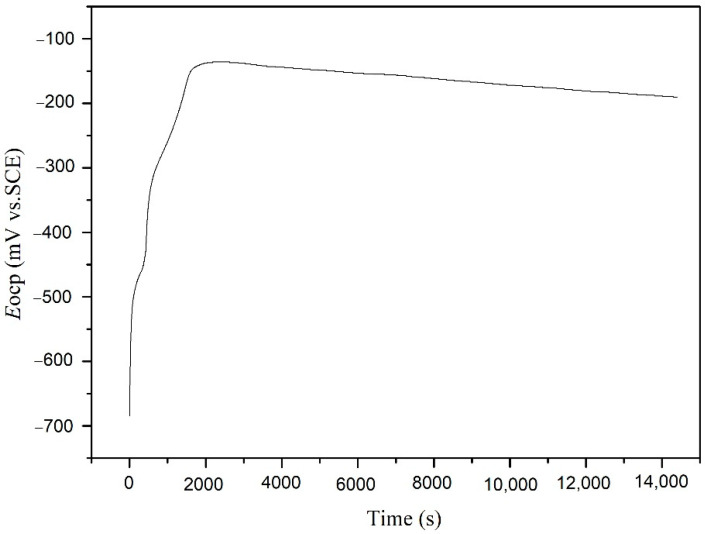
Open circuit potential of the carbon steel electrode immersed in NSCS for 21 days after depolarization.

The open circuit potential of the carbon steel electrode immersed in NSCS for 21 d after depolarization is shown in Figure 11. The potential values of the carbon steel electrode were −684.1, −142.7, −157.3, −174.9, and −190.4 mV after depolarization for 0, 1, 2, 3, and 4 h, respectively. The potential value of the carbon steel in concrete with conventional cathodic protection is usually between −763 mV and −1143 mV [49]. The received potential value of carbon steel was higher than that under conventional cathodic protection, as the power of the cement-based thermoelectric devices was less than that of conventional cathodic protection devices. The potential was more positive than −275 mV after depolarization for 4 h, indicating that the passive film on the surface of carbon steel was intact and no corrosion occurred on the surface of the carbon steel according to ASTM C876–2009. The potential attenuation was 493.7 mV after depolarization for 4 h, indicating that the cathodic protection system of the cement-based thermoelectric device can cause a negative potential shift of carbon steel and offer sufficient protection in the corrosion liquid according to NACE RP0169–96 and BS 7361–1–1991.

### 3.4. Electrochemical Impedance Spectroscopy (EIS)

**Figure 12:**
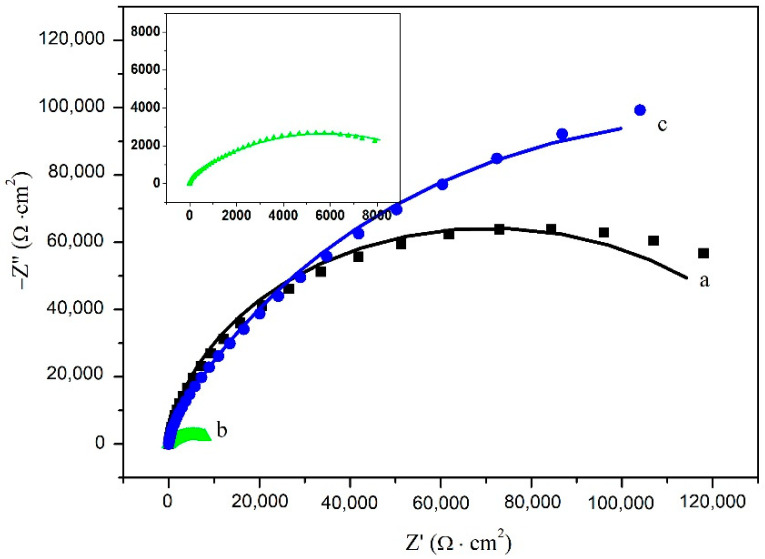
Nyquist plots of the carbon steel without cathodic protection (a: 0 day; b: 21 days) and with cathodic protection (c: 21 days) immersed in NSCS.

**Figure 13:**
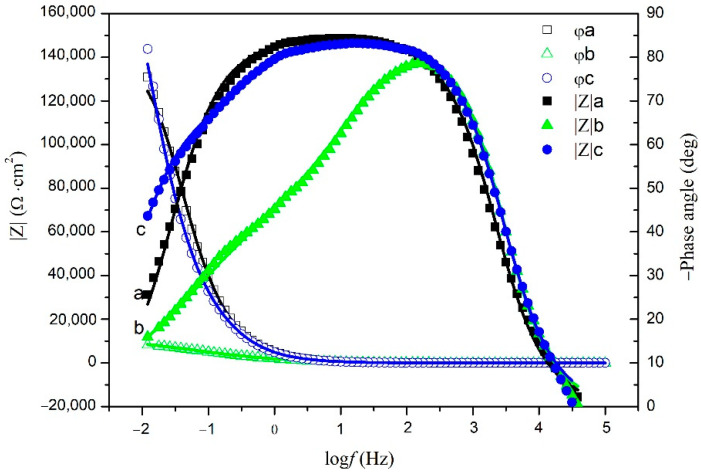
Bode plots of the carbon steel without cathodic protection (a: 0 day; b: 21 days) and with cathodic protection (c: 21 days) immersed in NSCS.

**Figure 14:**
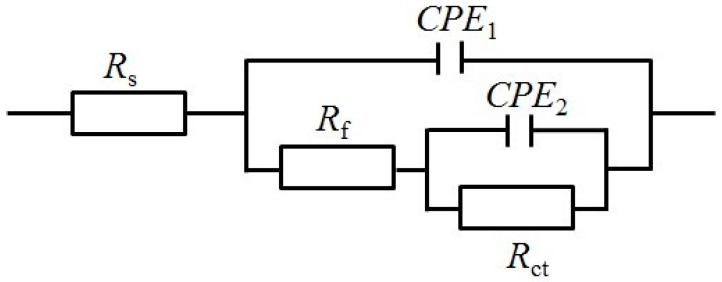
Equivalent circuit model used to fit EIS experiment data of carbon steel.

The electrochemical impedance plots of the carbon steel electrodes without and with cathodic protection immersed in NSCS for 0 and 21 d are shown in Figure 12 and Figure 13. The Nyquist plots show that the capacitive reactance arc of carbon steel in the high-frequency region is squashed, indicating that the corrosion reaction process is controlled by charge transfer resistance [50]. The equivalent circuit used to fit the EIS data of carbon steel is shown in Figure 14 [51,52]. The fitting results are shown in Table 3, where ***R*s** represents the solution resistance, ***R*_f_** represents the film resistance of the carbon steel surface, and ***R*ct** represents the charge transfer resistance in the corrosion reaction. *CPE*_1_ is composed of the film capacitance ***C*_f_**, and the dispersion coefficient ***n*_1_** represents the constant phase angle element. *CPE*_2_ is composed of a double electric layer capacitor ***C*_dl_**, and the dispersion coefficient ***n*_2_** represents the constant phase angle element.

The corrosion tendency of carbon steel can be analysed by ***R*_ct_** and ***R*_f_**. ***R*_ct_** of the carbon steel without cathodic protection decreased rapidly, indicating that the corrosion was quite serious at 21 days [53]. ***R*_ct_** of the carbon steel with cathodic protection increased rapidly, indicating that the carbon steel was effectively protected by the cement-based thermoelectric device [53]. In addition, ***R*_f_** of the carbon steel with cathodic protection was approximately 100 times that of the carbon steel without cathodic protection at 21 days. This reveals that the passive film on the carbon steel surface was strengthened after cathodic protection [54,55,56]. Therefore, the cathodic current generated by the cement-based thermoelectric device proved effective for the protection of carbon steel.

### 3.5. Polarization Potentiodynamics (PP)

**Figure 15:**
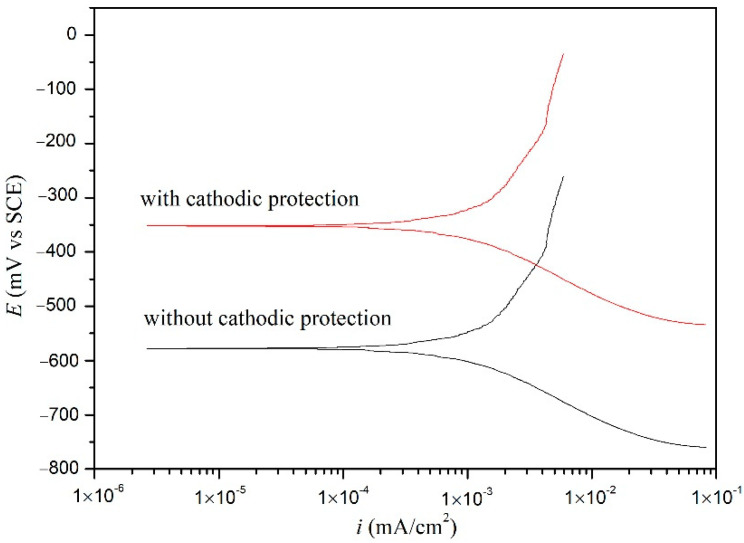
Polarization curves of the carbon steel without and with cathodic protection immersed in NSCS for 21 days.

Polarization curves of the carbon steel without and with cathodic protection immersed in NSCS for 21 d are shown in Figure 15. It can be observed that the location of the polarization curves moved toward the current reduction and potential increase direction when cathodic protection was applied on the carbon steel electrode. This reveals that both the cathode and anode reaction corrosion rates are reduced [51,55,56]. The calculated values of the electrochemical parameters, such as corrosion potential (*E*_corr_), corrosion current density (*i*_corr_), anodic Tafer slope (*β*_a_), and cathodic Tafer slope (*β*_c_) from the polarization measurements are listed in Table 4. The *i*_corr_ values of the carbon steel without and with cathodic protection were 1.67 and 0.32 μA/cm^2^, respectively. This revealed that the corrosion of carbon steel was delayed when the cathodic protection was applied. The corrosion current density of the carbon steel in concrete with conventional cathodic protection is usually between 0.1 to 0.2 μA/cm^2^ [52]. The received current density value of carbon steel was larger than that under conventional cathodic protection, as the power of the cement-based thermoelectric devices was less than that of conventional cathodic protection devices. The corrosion rate is high when the corrosion current density is greater than 1 μA/cm^2^, and it is low when the corrosion current density is less than 0.5 μA/cm^2^ [57]. Therefore, the cathodic protection provided by the cement-based thermoelectric device proved effective from polarization measurements.

To sum up, the carbon steel in alkaline chloride solution was effectively protected by the cement-based thermoelectric device according to the OCP, EIS, and PP experiments, although the anti-corrosion effect was poorer than conventional cathodic protection with impressed current. It provides a new method for protecting reinforced concrete in the simulated environment; however, it is currently far from practical application.

## 4. Conclusions

We designed and fabricated a cement-based thermoelectric device based on PANI/MnO_2_ composite for the protection of carbon steel in alkaline chloride solution. From the received results, the following conclusions can be drawn:

1. The PANI is deposited on the surface of the MnO_2_ particles, providing the PANI/MnO_2_ composite with good electrical conductivity because of the decrease in the electron conduction path, thereby increasing the electrical conductivity of cement composites with added PANI/MnO_2_ composite.
2. The nanostructured MnO_2_ particles provided the PANI/MnO_2_ composite with large Seebeck coefficient, because of the enhanced gradient of the density of states relative to the energy near the Fermi energy, thereby increasing the Seebeck coefficient of cement composites.
3. The decreased open circuit potential, increased charge transfer resistance, and decreased corrosion circuit density demonstrated that the carbon steel immersed in NSCS was effectively protected by the cement-based thermoelectric device.
4. In the light of the protection effect, the cement-based thermoelectric-device-driven cathodic protection for reinforced concrete structures suffering chloride corrosion becomes possible.

## Figures and Tables

**Table 1:** Composition of cement paste mixtures.

Mixture	Cement (g)	Fly Ash (g)	Water (g)	Superplasticizer (g)	PANI/MnO_2_ (g)	PANI/MnO_2_ Fraction (%)
M1	300	200	150	3.0	0	0
M2	300	200	150	3.0	5	1
M3	300	200	150	3.0	10	2
M4	300	200	150	3.0	15	3
M5	300	200	150	3.0	20	4
M6	300	200	150	3.0	25	5

**Table 2:** PANI content and MnO_2_ content in the prepared PANI/MnO_2_ composite.

PANI/ MnO_2_ Composite	TGA Curve	MnO_2_ Dosages (g)	PANI Content (wt.%)	MnO_2_ Content (wt.%)	MnO_2_ Content (vol.%)
C1	b	4.0	26.5	73.5	30.7
C2	c	6.0	22.3	77.7	35.9
C3	d	8.0	11.6	88.4	55.0
C4	e	10.0	10.0	90.0	59.1
C5	f	15.0	7.3	92.7	67.1

**Table 3:** Electrochemical parameters calculated from the EIS data of carbon steel immersed in NSCS without and with cathodic protection.

Series	Immersion Time (Day)	*R_S_* (Ω·cm^2^)	*C_f_* (μF·cm^−2^)	*n*_1_	*R_f_* (Ω·cm^2^)	*C_dl_* (μF·cm^−2^)	*n*_2_	*R_ct_* (kΩ·cm^2^)
Without cathodic protection	0	3.60	15.66	0.990	7.33	20.53	0.872	1.46 × 10^2^
	21	3.00	27.60	0.960	5.16 × 10^2^	175.1	0.509	11.4
With cathodic protection	21	2.84	35.35	0.938	5.14 × 10^4^	25.87	0.768	1.98 × 10^6^

**Table 4:** Polarization parameters for carbon steel specimens in corrosion solution.

Cathodic Protection	*E*_corr_ (mV)	*β*_c_ (mV/dec)	*β*_a_ (mV/dec)	*i*_corr_ (μA/cm^2^)
No	−522.1	−128	303	1.67
Yes	−325.5	−133	217	0.32

## Data Availability

The data presented in this study are available on request from the corresponding author.

## References

[1] Marcos-Meson V., Michela A., Solgaard A., Fischer G., Edvardsen C., Skovhus T.L. (2018). Corrosion resistance of steel fibre reinforced concrete—A literature review. Cem. Concr. Res..

[2] Liu X., Wang J., Hu W. (2021). Facile synthesis of novel hierarchical core@ shell structural magnetic nanovehicle Fe_3_O_4_@ ZnAlCe-MoO_4_-LDHs for carbon steel protection. J. Magn. Magn. Mater..

[3] Feng R., Zhang J.Z., Zhu J.H., Xing F. (2020). Experimental study on the behavior of carbon-fabric reinforced cementitious matrix composites in impressed current cathodic protection. Constr. Build. Mater..

[4] Ackland B.G., Dylejko K.P. (2020). Critical questions and answers about cathodic protection. Corros. Eng. Sci. Technol..

[5] Oleiwi H.M., Wang Y., Curioni M., Chen X.Y., Yao G.W., Augusthus-Nelson L., Ragazzon-Smith A.H., Shabalin I. (2018). An experimental study of cathodic protection for chloride contaminated reinforced concrete. Mater. Struct..

[6] Hassanein A.M., Glass G.K., Buenfeld N.R. Intermittent cathodic protection of concrete structures. Proceedings of the Fourth International Symposium.

[7] Christodoulou C., Glass G., Webb J., Austin S., Goodier C. (2010). Assessing the long term benefits of Impressed Current Cathodic Protection. Corros. Sci..

[8] Yezhov V., Semicheva N., Pakhomova E., Burtsev A., Brezhnev A., Perepelitsa N. Characterization of thermoelectric generators for cathodic protection of pipelines of the city heating. Proceedings of the International Scientific Conference Energy Management of Municipal Facilities and Sustainable Energy Technologies EMMFT 2018.

[9] Sun M., Li Z., Mao Q., Shen D. (1998). Thermoelectric percolation phenomenon in carbon fiber-reinforced concrete. Cem. Concr. Res..

[10] Wen S., Chung D.D.L. (2000). Enhancing the Seebeck effect in carbon fiber reinforced cement by using intercalated carbon fibers. Cem. Concr. Res..

[11] Demirel B., Yazicioglu S. (2008). Thermoelectric behavior of carbon fiber reinforced lightweight concrete with mineral admixtures. New Carbon Mater..

[12] Wei J., Zhang Q., Zhao L., Hao L., Yang C.L. (2016). Enhanced thermoelectric properties of carbon fiber reinforced cement composites. Ceram. Int..

[13] Zuo J., Yao W., Liu X., Qin J.J. (2012). Sensing properties of carbon nanotube-carbon fiber/cement nanocomposites. J. Test. Eval..

[14] Tzounis L., Liebscher M., Fuge R., Leonhardt A., Mechtcherine V. (2019). P- and n-type thermoelectric cement composites with CVD grown p- and n-doped carbon nanotubes: Demonstration of a structural thermoelectric generator. Energy Build..

[15] Wei J., Fan Y., Zhao L., Xue F., Hao L., Zhang Q. (2018). Thermoelectric properties of carbon nanotube reinforced cement-based composites fabricated by compression shear. Ceram. Int..

[16] Ghosh S., Harish S., Rocky K.A., Ohtaki M., Saha B.B. (2019). Graphene enhanced thermoelectric properties of cement based composites for building energy harvesting. Energy Build..

[17] Ghosh S., Harish S., Ohtaki M., Saha B.B. (2020). Enhanced figure of merit of cement composites with graphene and ZnO nanoinclusions for efficient energy harvesting in buildings. Energy.

[18] Wei J., Zhao L., Zhang Q., Nie Z.B., Hao L. (2018). Enhanced thermoelectric properties of cement-based composites with expanded graphite for climate adaptation and large-scale energy harvesting. Energy Build..

[19] Wei J., Hao L., He G., Yang C.L. Thermoelectric power of carbon fiber reinforced cement composites enhanced by Ca_3_Co_4_O_9_. Proceedings of the China Functional Materials Technology and Industry Forum.

[20] Wei J., Hao L., He G., Yang C.L. (2014). Enhanced thermoelectric effect of carbon fiber reinforced cement composites by metallic oxide/cement interface. Ceram. Int..

[21] Ghaharia S., Ghafaria E., Lu N. (2017). Effect of ZnO nanoparticles on thermoelectric properties of cement composite for waste heat harvesting. Constr. Build. Mater..

[22] Ji T., Zhang X., Li W.H. (2016). Enhanced thermoelectric effect of cement composite by addition of metallic oxide nanopowders for energy harvesting in buildings. Constr. Build. Mater..

[23] Ji T., Zhang X.Y., Zhang X., Zhang Y.J., Li W.H. (2018). Effect of manganese dioxide nanorods on the thermoelectric properties of cement composites. J. Mater. Civ. Eng..

[24] Pichanusakorn P., Bandaru P. (2010). Nanostructured thermoelectrics. Mater. Sci. Eng. R Rep..

[25] Lan Y., Minnich A.J., Chen G., Ahn J.P., Sung Y.M. (2010). Enhancement of thermoelectric figure-of-merit by a bulk nanostructuring approach. Adv. Funct. Mater..

[26] Alam H., Ramakrishna S. (2013). A review on the enhancement of figure of merit from bulk to nano-thermoelectric materials. Nano Energy.

[27] Tong T., Fu D., Levander A.X., Schaff W.J., Pantha B.N., Lu N., Liu B., Ferguson I., Zhang R., Lin J.Y. (2013). Suppression of thermal conductivity in InxGa1−xN alloys by nanometer-scale disorder. Appl. Phys. Lett..

[28] Chen B., Wu K., Yao W. (2004). Conductivity of carbon fiber reinforced cement-based composites. Cem. Concr. Compos..

[29] Belli A., Mobili A., Bellezze T., Tittarelli F. (2020). Commercial and recycled carbon/steel fibers for fiber-reinforced cement mortars with high electrical conductivity. Cem. Concr. Compos..

[30] Zhang H., Wu X.H., Wang X.L. (2011). Conductivity mechanism of asphalt concrete with the PANI/PP compound conductive fiber. Mater. Sci. Forum.

[31] Meng Q., Chung D.D.L. (2010). Battery in the form of a cement-matrix composite. Cem. Concr. Compos..

[32] Azarsa P., Gupta R. (2017). Electrical resistivity of concrete for durability evaluation: A review. Adv. Mater. Sci. Eng..

[33] Pani L., Francesconi L., Rombi J., Mistretta F., Sassu M., Stochino F. (2020). Effect of parent concrete on the performance of recycled aggregate concrete. Sustainability.

[34] Feng C., Huang J., Yan P., Wan F., Zhu Y., Cheng H. (2021). Preparation and properties of waterborne polypyrrole/cement composites. Materials.

[35] Jiang S., Zhou D., Zhang L., Ouyang J., Yu X., Cui X., Han B. (2018). Comparison of compressive strength and electrical resistivity of cementitious composites with different nano- and micro-fillers. Arch. Civ. Mech. Eng..

[36] Fu C., Xie C., Liu J., Wei X., Wu D. (2020). A Comparative Study on the effects of three nano-materials on the properties of cement-based composites. Materials.

[37] Naveen A.N., Selladurai S. (2015). Fabrication and performance evaluation of symmetrical supercapacitor based on manganese oxide nanorods–PANI composite. Mater. Sci. Semicon. Proc..

[38] Tarmizi A.A.A., Harun M.K., Lyana S.N. (2021). Effect of modified nano silica on the conductivity and morphology characteristics of oxalic acid-doped polyaniline composites. Int. J. Electrochem. Sci..

[39] Roy H.S., Islam M.M., Mollah M.Y.A., Susan M.A.B.H. (2020). Polyaniline-MnO_2_ composites prepared in-situ during oxidative polymerization of aniline for supercapacitor applications. Mater. Today.

[40] Li Y., Xu Z.Y., Wang D.W., Zhao J., Zhang H.H. (2017). Snowflake-like core-shell α-MnO_2_@ δ-MnO_2_ for high performance asymmetric supercapacitor. Electrochim. Acta.

[41] Mu Y.K., Ruan C.H., Li P.X., Xu J., Xie Y.B. (2020). Enhancement of electrochemical performance of cobalt (II) coordinated polyaniline: A combined experimental and theoretical study. Electrochim. Acta.

[42] Zhang J., Shu D., Zhang T., Chen H., Zhao H., Wang Y., Sun Z., Tang S., Fang X., Cao X. (2012). Capacitive properties of PANI/MnO_2_ synthesized via simultaneous-oxidation route. J. Alloys Compd..

[43] Jiang H., Ma J., Li C. (2012). Polyaniline–MnO_2_ coaxial nanofiber with hierarchical structure for high-performance supercapacitors. J. Mater. Chem..

[44] Wei J., Jia Z., Wang Y., Jiang Y., Miao Z., Zhou Y., Zhang H. (2021). Enhanced thermoelectric performance of low carbon cement-based composites by reduced graphene oxide. Energy Build..

[45] Wei J., Wang Y., Li X., Jia Z., Qiao S., Jiang Y., Zhou Y., Miao Z., Gao D., Zhang H. (2021). Dramatically improved thermoelectric properties by defect engineering in cement-based composites. ACS Appl. Mater. Interfaces.

[46] Ghosh S., Harish S., Ohtaki M., Saha B.B. (2021). Thermoelectric figure of merit enhancement in cement composites with graphene and transition metal oxides. Mater. Today Energy.

[47] Carraro P.A., Maragoni L., Paipetis A.S., Quaresimin M., Tzounis L., Zappalorto M. (2021). Prediction of the Seebeck coefficient of thermoelectric unidirectional fibre-reinforced composites. Compos. Part B.

[48] Liu X., Liao G., Zuo J. (2021). Enhanced thermoelectric properties of carbon fiber-reinforced cement composites (CFRCs) utilizing Bi_2_Te_3_ with three doping methods. Fuller. Nanotub. Carbon Nanostruct..

[49] Cubides Y., Castaneda H. (2016). Corrosion protection mechanisms of carbon nanotube and zinc-rich epoxy primers on carbon steel in simulated concrete pore solutions in the presence of chloride ions. Corros. Sci..

[50] Zoltowski P. (1998). On the electrical capacitance of interfaces exhibiting constant phase element behavior. J. Electron. Chem..

[51] Ji T., Ma F., Liu D., Zhang X., Zhang X., Luo Q. (2018). Effect of diamino ((2-((2-aminoethyl) amino) ethyl) amino) methanethiol on the corrosion resistance of carbon steel in simulated concrete pore solutions. Int. J. Electrochem. Sci..

[52] Jin M., Gao S., Jiang L., Jiang Y., Wu D., Song R., Wu Y., He J. (2018). Continuous monitoring of steel corrosion condition in concrete under drying/wetting exposure to chloride solution by embedded MnO_2_ sensor. Int. J. Electrochem. Sci..

[53] Wang C.L., Gao W., Liu N.Z., Xin Y., Liu X.Y., Wang X.T., Tian Y., Chen X.W., Hou B.R. (2020). Covalent organic framework decorated TiO_2_ nanotube arrays for photoelectrochemical cathodic protection of steel. Corros. Sci..

[54] Brownlie F., Giourntas L., Hodgkiess T., Palmeira I., Odutayo O., Galloway A.M., Pearson A. (2020). Effect of cathodic protection methods on ferrous engineering materials under corrosive wear conditions. Corros. Eng. Sci Technol..

[55] Jiang X.H., Sun M.M., Chen Z.Y., Jing J.P., Feng C. (2020). High-efficiency photoelectrochemical cathodic protection performance of the TiO_2_/AgInSe_2_/In_2_Se_3_ multijunction nanosheet array. Corros. Sci..

[56] Lu Y., Hu J., Li S., Tang W. (2018). Active and passive protection of steel reinforcement in concrete column using carbon fibre reinforced polymer against corrosion. Electrochim. Acta.

[57] Andrade C., Alonso C. (2004). Test methods for on-site corrosion rate measurement of steel reinforcement in concrete by means of the polarization resistance method. Mater. Struct..

